# Novel Optical Technology in Bladder Cancer Diagnosis and Treatment

**DOI:** 10.5812/numonthly.16363

**Published:** 2014-01-23

**Authors:** Athanasios Dellis, Athanasios Papatsoris

**Affiliations:** 1 Department of Urology, School of Medicine, Sismanoglio Hospital, University of Athens, Athens, Greece; 2 Department of Surgery, School of Medicine, Areteion Hospital, University of Athens, Athens, Greece

**Keywords:** Optical Imaging, Cystoscopy, Urinary Bladder

Bladder cancer is diagnosed and followed-up with cystoscopy as the “gold standard”. For many decades white light cystoscopy (WLC) has been used. However, several forms of bladder cancer such as the high-risk carcinoma in situ (CIS) may be misdiagnosed with WLC. As WLC still remains the cornerstone for the diagnosis of bladder cancer progress in endoscopic technology is warranted to improve the diagnostic accuracy of both rigid and flexible cystoscopy.

Recently, photodynamic diagnosis (PDD) has been introduced for the diagnosis and transurethral resection of bladder cancer (TURBT), especially in cases of CIS (1). Photo-sensitizing drugs are intravesically administrated and are selectively accumulated in cancer cells, leading to the enhancement of contrast between benign and malignant bladder tissues. Prospective randomized studies have demonstrated that PDD improved bladder cancer detection, reduced residual tumor rates after TURBT, and prolonged the recurrence-free survival (1, 2). These studies have demonstrated the superiority of PDD-guided cystoscopy over WLC in tumor detection, as the sensitivity for PDD was 76-97% compared to 46–80% for WLC (1, 2).

Narrow band imaging (NBI) is a novel technique used in endoscopy to enhance tissue contrast between cancerous and normal bladder urothelium, while it does not require the administration of contrast agents (1). This technique is based on the phenomenon that the depth of light penetration into the urothelium increases when the wavelength is increased. In particular, NBI uses a light source that filters white light into two wavelengths (415 and 540 nm) which are strongly absorbed by hemoglobin, enhancing the contrast between subepithelial capillaries and mucosa (3). Prospective studies and meta-analyses have demonstrated that 10-25% cases of bladder cancer missed by WLC were detected by NBI (1, 4). Furthermore, NBI resulted in lower rates of residual bladder tumor during repeat TURBT and in reduced recurrence rates (4, 5).

Optical coherence tomography (OCT) measures the backscattering of near-infrared light by tissue, and it yields in 2D and 3D images at micrometer-scale resolution, thus providing optical biopsies, approaching the resolution of histopathological imaging (6). It produces high-resolution, cross-sectional images of the bladder tissue enabling real-time bladder cancer staging (6). The principle of OCT is analogous to the B-mode ultrasound except that light is being used instead of sound. Studies have demonstrated that OCT results in high sensitivity and specificity rates for bladder cancer diagnosis (6, 7). The usage of relevant algorithms (i.e. Lingley-Papadopoulos) contributes to the differentiation of benign and malignant bladder tissue with a sensitivity rate of 92 % and a specificity rate of 62 % (7).

Raman spectroscopy (RS) is a novel spectroscopic optical technique based on the phenomenon of proton emission after light and tissue molecules interaction. Studies have demonstrated that it successfully diagnosed bladder cancer with sensitivity and specificity rates of 85% and 79%, respectively (8). Similarly, confocal laser endomicroscopy (CLE) is an emerging technology based on the established principles of confocal microscopy, which enables in vivo real-time imaging of the tissue micro-architecture and cellular morphology with promising results. In vivo probe-based CLE of the bladder demonstrated distinct differences between normal mucosa and neoplastic tissues and by using mosaicing, a post hoc image-processing algorithm, individual image frames were juxtaposed to form wide-angle views to better evaluating tissue microarchitecture (9). Lastly, wireless capsule endoscopy (WCE) has been successfully used in Gastroenterology since the 1990s. Recently, it was successfully evaluated in a pig model where the ability to deploy and manipulate the capsule within the bladder was examined as well as the feasibility of capturing and retrieving images in real time (10).

In conclusion, novel optical imaging technologies have emerged showing promising results for the diagnosis, management and follow up of patients with bladder cancer. The results of adequately powered comparative studies are warranted to determine the efficacy of these novel techniques in comparison to the standard WLC.
